# Supplementary material for: Association of Urinary Lead and Cadmium Levels, and Serum Lipids with Subclinical Arteriosclerosis: Evidence from Taiwan
Source: Nutrients. 2023 Jan 21;15(3):571. doi: 10.3390/nu15030571 (PMC9919350; doi:10.3390/nu15030571)
Supplement: Supplementary file 1 [file nutrients-15-00571-s001.zip › nutrients-2136788-supplementary.pdf]

Supplementary material

Association of Urinary Lead and Cadmium Levels, and Serum Lipids with Subclinical Arteriosclerosis: Evidence from Taiwan

Chien-Yu Lin,[1,2,3] Hui-Ling Lee,[4] Yi-Ting Hwang,[5] Po-Chin Huang,[6,7] Fung-Chang Sung,[8] and Ta-Chen Su[9,10,11*]

[1]Department of Internal Medicine, En Chu Kong Hospital, New Taipei City 237, Taiwan

[2]School of Medicine, Fu Jen Catholic University, New Taipei City 242, Taiwan

[3]Department of Environmental Engineering and Health, Yuanpei University of Medical Technology, Hsinchu 300, Taiwan

[4]Department of Chemistry, Fu Jen Catholic University, New Taipei City 242, Taiwan

[5]Department of Statistics, National Taipei University, New Taipei City 237, Taiwan

[6]National Institute of Environmental Health Sciences, National Health Research Institutes, Miaoli 350, Taiwan

[7]Department of Medical Research, China Medical University Hospital, China Medical University, Taichung 404, Taiwan

[8]Department of Health Services Administration, College of Public Health, China Medical University, Taichung 404, Taiwan

[9]Department of Environmental and Occupational Medicine, National Taiwan University Hospital, Taipei 100, Taiwan

[10]Department of Internal Medicine and Cardiovascular Center, National Taiwan University Hospital, Taipei 100, Taiwan

[11]Institute of Environmental and Occupational Health Sciences, College of Public Health, National Taiwan University, Taipei, 100, Taiwan

File S1 Supplemental methods

Study population and data collection

From 1992 to 2000, approximately 2,615,000 to 2,932,000 school-age children in grades 1 to 12 received an annual urine screening of urine strip by the Chinese Foundation of Health in Taipei, Taiwan. Subjects with abnormal results from two tests for proteinuria, glycosuria, or hematuria underwent a third urine screening test and a general health check-up. A total of 103,756 school children received the health check-ups and the third urine screen. According to the American Heart Association, childhood hypertension is defined as a systolic blood pressure at or above the 90th percentile and diastolic blood pressure should be at or above the 95th percentile for children of the same age, sex, and height. Among these children, 9,227 had elevated blood pressure and 94,529 had normal blood pressure.

From 2006 to 2008 we established a cohort, the YOung TAiwanese Cohort (YOTA) study, based on students with and without childhood EBP, selected from the 1992–2000 urine mass screening population. In the follow-up, we mailed invitation letters to eligible students in the Taipei area. After 3–5 days, 12 trained assistants and nurses conducted telephone interviews inviting those subjects with childhood EBP to come in for a follow-up health examination. No telephone interview contact was made with normotensive students. Among the 707 subjects with EBP in childhood, 303 completed the follow-up health examinations, giving a response rate of 42.9%. Among the 6,390 subjects with normal BP in childhood, 486 completed the follow-up health examinations, giving a response rate of 7.6%. In order to differentiate the effects of environment on age of exposure, we recruited 97 subjects as “best friend controls” in the cohort follow-up period. A total of 886 subjects were included in this study. Physical check-ups were given after written informed consent. All methods in this study were performed in accordance with the relevant guidelines and approved by the Research Ethics Committee of the National Taiwan University Hospital. A flow chart of this

study is shown in Figure 1. Among 886 participants, 148 individuals were excluded because of unavailability of urine samples for testing. Finally, 738 participants were enrolled in the present study.

Measurement of urinary lead and cadmium levels

Collected urine specimens were kept in a freezer (-20°C) until shipment and then immediately stored at −80°C. After thawing the urine samples, 1 mL of the samples were diluted 10-fold with 9 mL of 1% (v/v) nitric acid (J.T. Baker Chemical Company, Phillipsburg, NJ, USA) in 15 mL polypropylene tubes and analyzed by inductively coupled plasma-mass spectrometry (ICP-MS, 7700 series; Agilent Technologies, Inc., Santa Clara, CA, USA).

A guideline of quality control and method detection limit (MDL) were modified from the National Institute of Environmental Analysis PA-103, PA-104, and PA-107, Taiwan EPA (NIEA PA-103, PA-104, PA-107, 2005), which is based on the standards of the National Environmental Laboratory Accreditation. The calibration curves ranged from 0.01 to 50 μg/L for lead and cadmium (SPEX CertiPrep; Multi-element Solution 2, CLMS-2, Metuchen, NJ, USA) with correlation coefficient [R^2] >0.995. To ensure the quality of analysis results, one sample of blank, spiked, duplicate, and quality control was tested in every batch of 10 samples. The concentration of lead and cadmium in the blank sample was less than two-fold of the MDL, while the recoveries of 1 μg/L spiked urine and 5 μg/L quality control samples were within ±20%, Each sample was run in duplicate within ±10% precision. If lead in urine sample was below MDL (Lead : 0.007 μg/L, Cadmium: 0.006 μg/L), it would be replaced by one-half the MDL.

Measurement of carotid intima-media thickness (CIMT)

The measurement of CIMT is determined by the distance from the leading edge of the

first echogenic line (i.e., the boundary between the lumen and the inner lining of the vessel) to the leading edge of the second echogenic line (i.e., the boundary between the middle layer of the vessel and the outer layer) in the far wall of the vessel. An experienced technician used a high-resolution B-mode ultrasonography (GE Vivid ultrasound system, Horten, Norway) equipped with a 3.5–10 MHz real-time B-mode scanner to examine the CIMT of extracranial carotid arteries; we then applied a software package for vascular ultrasound for off-line automatic calculations. The CIMTs of the common carotid artery (CCA) proximal to the carotid bifurcation, bulb, and internal carotid artery (ICA) were obtained bilaterally. CCA1 and CCA2 are specific point located 0–1 cm and 1–2 cm, respectively, along the CCA distal to the carotid bifurcation. We averaged CCA1 and CCA2 to obtain a mean that is representative of the CCA. Mean CIMT in this analysis was determined by averaging four measurements on bilateral CCAs. To determine the reliability of repeated measurements, the technician conducted a second reading on 30 randomly selected subjects 2 weeks later. The reliability of the CIMT measurement from bilateral CCAs (i.e., the mean of the right and left CCA) had excellent intra-observer coefficient of correlation reliability (ICCR), approximately 98.8% and 98.5%, respectively.

Covariates

Demographic data were collected during the interview. Household income was categorized as above or below 50,000 New Taiwan Dollars (NTD) per month. Alcohol consumption was divided into current alcohol consumption or not. Smoking status was divided into not active smokers and active smokers. The classification of exercise was whether there is an exercise habit now. Dietary sweets were classified as eating sweets ≥ or < five times per week. Dietary fat was classified as eating fatty meat and/or fast food combined ≥ or < six times a week. The body mass index (BMI) was calculated as weight in kilograms

divided by height in meters squared. For participations ≥ 20 year-old, BMI z-score was measured by equation (BMI of each participants - mean of BMI)/(standard deviation of BMI) while aged 12-19 years were calculated based on WHO anthropometric calculator. Blood pressure was measured twice after 3 min of rest using a mercury manometer. In adults, hypertension was defined as the self-reported current use of anti-hypertensive medication or either average systolic blood pressure ≥ 140 mmHg or average diastolic blood pressure ≥ 90 mmHg. Childhood hypertension was determined by blood pressure values ≥ the modified sex- and age-specific criteria. Diabetes mellitus was defined as a fasting serum glucose level ≥126 mg/dL or current use of medications to treat hyperglycemia. Serum insulin concentrations were quantified by the kit IMMULITE 2000 (Siemens Healthcare Diagnostics, Tarrytown, NY). The homeostasis model assessment of insulin resistance index was measured by the product of basal glucose and insulin levels divided by 22.5.

Supplemental Table S1. Demographic data and metal exposure variables in the studied Taiwanese population (continuous variables) (n = 736).

	Mean (SD)
Systolic blood pressure (mmHg)	107.44 (14.42)
HOMAR-IR	1.28 (1.87)
Lipid profiles (mg/dL)	
LDL-C	81.28 (30.55)
sdLDL-C	15.49 (10.74)
LDL-TG	9.20 (18.83)
HDL-C	50.22 (9.86)
Lipoprotein(a)	9.00 (9.95)
Apolipoprotein A1	136.86 (18.19)
Apolipoprotein B	77.05 (18.03)
Triglyceride	83.92 (77.13)
Urine heavy metals	
Lead (μg/g creatinine)	7.52 (18.99)
Lead (μg/L)	9.41 (17.85)
Cadmium (μg/g creatinine)	1.49 (2.79)
Cadmium (μg/L)	2.02 (2.77)
CIMT (μm)	447.04 (53.89)

Abbreviations: BMI: body mass index; HOMA-IR: homeostasis model assessment of insulin resistance; HDL-C: high-density lipoprotein cholesterol; LDL-C: low-density lipoprotein cholesterol; LDL-TG: low-density lipoprotein triglyceride; sdLDL-C: small dense low-density lipoprotein cholesterol.

Supplemental Table S2. Linear regression coefficients (standard error) of CIMT (μm) with a unit increase in lipid profiles (mg/dL) in multiple linear regression models (n = 866).

	CIMT	
	Adjusted β (SE)	P
LDL-C	0.426 (0.064)	<0.001
sdLDL-C	1.034 (0.187)	<0.001
LDL-TG	0.061 (0.103)	0.558
HDL-C	-0.304 (0.218)	0.164
Lipoprotein(a)	0.045 (0.192)	0.813
Apolipoprotein A1	0.100 (0.113)	0.378
Apolipoprotein B	0.402 (0.110)	<0.001
Triglyceride	-0.011 (0.027)	0.690

Model: adjusted for age, gender, BMI z score, smoking status, drinking status, exercise, and household income, dietary sweets, dietary fat, systolic blood pressure, HOMA-IR.

Abbreviations: BMI: body mass index; HOMA-IR: homeostasis model assessment of insulin resistance; HDL-C: high-density lipoprotein cholesterol; LDL-C: low-density lipoprotein cholesterol; LDL-TG: low-density lipoprotein triglyceride; sdLDL-C: small dense low-density lipoprotein cholesterol.

Supplemental Table S3: Linear regression coefficients (standard error) of CIMT (μm) per unit increase in ln-heavy metal concentration (μg/g creatinine) by different categories of lipoprotein concentrations (mg/dL) in separate and composite analysis.

		Pb		Cd	
Separate analysis		Adjusted *β* (SE)	*P*	Adjusted *β* (SE)	*P*
LDL-C	Total	14.939 (1.053)	<0.001	18.067 (1.418)	<0.001
	< 76.5 (< 50%ile)	10.873 (1.402)	<0.001	12.337 (1.817)	<0.001
	≥ 76.5 (≥ 50%ile)	16.182 (1.610)	<0.001	20.323 (2.212)	<0.001
P for interaction			<0.001		0.092
sdLDL-C	Total	14.939 (1.053)	<0.001	18.067 (1.418)	<0.001
	< 13.0 (< 50%ile)	11.628 (1.410)	<0.001	13.154 (1.890)	<0.001
	≥ 13.0 (≥ 50%ile)	16.298 (1.617)	<0.001	20.060 (2.155)	<0.001
P for interaction			0.001		0.216
Composite analysis					
LDL-C	Total	11.793 (3.025)	<0.001	6.977 (3.966)	0.079
	< 76.5 (< 50%ile)	8.628 (2.309)	<0.001	3.597 (2.942)	0.222
	≥ 76.5 (≥ 50%ile)	11.962 (2.958)	<0.001	6.776 (3.989)	0.090
P for interaction			<0.001		0.088
sdLDL-C	Total	11.793 (3.025)	<0.001	6.977 (3.966)	0.079
	< 13.0 (< 50%ile)	9.790 (2.290)	<0.001	3.054 (2.998)	0.309
	≥ 13.0 (≥ 50%ile)	11.793 (3.025)	<0.001	6.977 (3.966)	0.079
P for interaction			0.002		0.171

Model: adjusted for age, gender, BMI z score, smoking status, drinking status, exercise, household income, dietary sweets, dietary fat, systolic blood pressure, HOMA-IR.

Abbreviations: BMI: body mass index; HOMA-IR: homeostasis model assessment of insulin resistance; HDL-C: high-density lipoprotein cholesterol; LDL-C: low-density lipoprotein cholesterol; LDL-TG: low-density lipoprotein triglyceride; sdLDL-C: small dense low-density lipoprotein cholesterol.
